# Injectional Anthrax in Heroin Users, Europe, 2000–2012

**DOI:** 10.3201/eid2002.120921

**Published:** 2014-02

**Authors:** Matthias Hanczaruk, Udo Reischl, Thomas Holzmann, Dimitrios Frangoulidis, David M. Wagner, Paul S. Keim, Markus H. Antwerpen, Hermann Meyer, Gregor Grass

**Affiliations:** Bundeswehr Institute of Microbiology, Munich, Germany (M. Hanczaruk, D. Frangoulidis, M.H. Antwerpen, H. Meyer, G. Grass);; University Hospital Regensburg, Regensburg, Germany (U. Reischl, T. Holzmann);; Northern Arizona University, Flagstaff, Arizona, USA (D. Wagner, P.S. Keim)

**Keywords:** Bacillus anthracis, anthrax, heroin users, single nucleotide polymorphism, Germany, Trans-Eurasian clade, phylogeny, bacteria, Europe, injectional anthrax, humans

**To the Editor:** Anthrax is a global zoonotic disease, but human infections are rare in countries of Western Europe. During 2009–2010, a total of 119 (47 laboratory-confirmed) drug-abuse–related cases of anthrax were reported in the United Kingdom and Germany (*1*). In these patients, the disease had an unusual manifestation. In contrast to acquiring the disease through typical routes of infection, leading to cutaneous, inhalation, or gastrointestinal anthrax, these patients became infected by injecting heroin (*1**–**3*). The term injectional anthrax was then coined to describe this new mode of infection. Patients with injectional anthrax show severe symptoms, and death rates are high. Of the 47 patients with confirmed cases of injectional anthrax acquired during the 2009–2010 outbreak, 19 died from the disease (*1*). However, this outbreak was not the first report of death caused by *B. anthracis* in an injectional drug (heroin) user; the disease was described in 1 person who died in Norway in 2000 (*3*).

Attempts to directly connect the United Kingdom cases to batches of anthrax-contaminated heroin were unsuccessful. No viable *B. anthracis* or DNA could be retrieved from the investigation’s drug samples. However, PCR- or culture-positive samples were obtained from some patients and used for genotyping (*2*). Later, researchers from Arizona and the United Kingdom worked together to use a high-resolution molecular approach to genotype the 2009–2010 outbreak strains. This information was used to obtain insight into the epidemiology and likely geographic origin of the European outbreak strains. 

All patients were infected by a single *B. anthracis* strain type (*2*,*4*) that belonged to the large Trans-Eurasian clade of *B. anthracis*. The whole genome of a representative strain of this type, Ba4599, had previously been sequenced (*2*). Strains related to strains associated with those isolated from European drug users, which belong to the A.Br. 008/011 canSNP cluster but are still genetically distinct, have so far only been identified from Turkey (*2*). However, more isolates from other relevant regions need to be investigated to confirm the likely geographic source.

In June 2012, after a 20-month gap, 2 new cases of injectional anthrax in heroin consumers were reported in Bavaria (*5*,*6*). Additional cases have been reported since then from Germany, Denmark, the United Kingdom, and France, leading to 26 deaths as of August 2013 (*7*). 

Molecular phylogenetic methods were used to determine the genetic relatedness of these strains with Ba4599 (*8*,*9*). Genotyping results using canonical single nucleotide polymorphisms (SNPs) (Figure) (*2*,*4*) placed all of these strains along branch A.Br.008 within the Trans-Eurasian group of *B. anthracis* (*10*). Further hierarchical fine-scale typing of the isolates by interrogating SNPs that were discovered from the heroin-associated strain Ba4599 (*2*) indicated that all isolates are identical at these SNPs (Figure) (*7*). The initial strain isolated in Norway in 2000 also falls into this group (*7*). In addition, analysis by multiple locus variable number tandem repeats suggested that all investigated strains are closely related, differing at just 2 markers (*7*). Thus, we conclude that all injectional anthrax isolates likely came from the same source.

**Figure:**
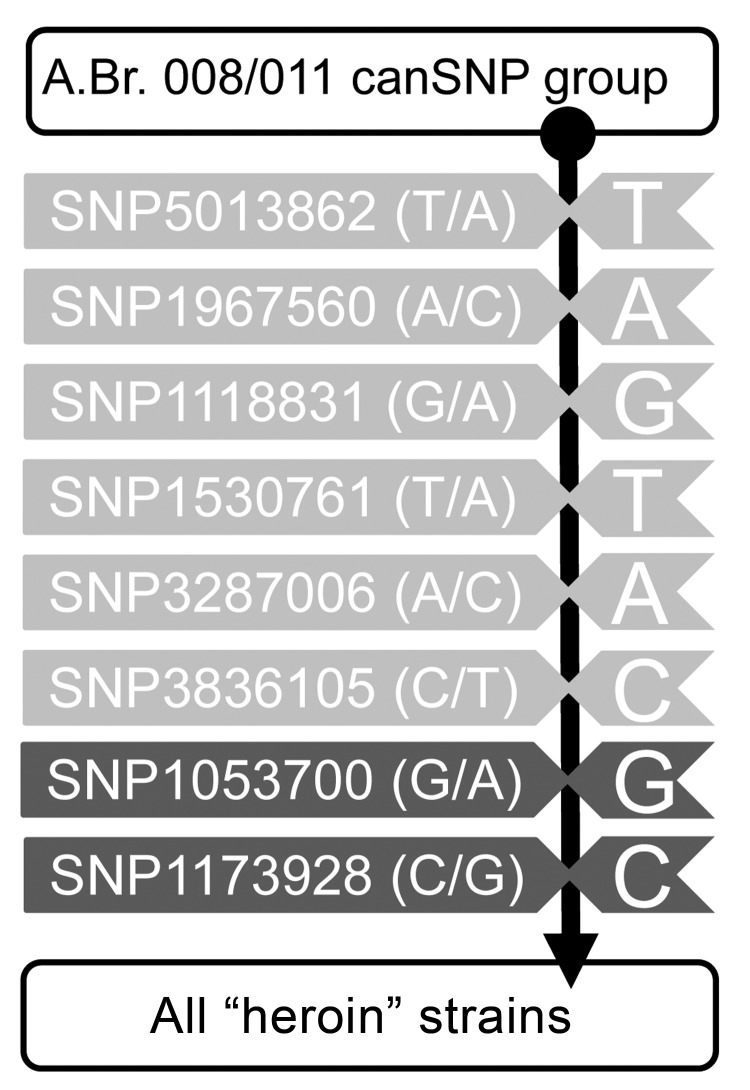
Diagram of single nucleotide polymorphism (SNP) assays used for bioforensic genotyping of heroin-associated *Bacillus anthracis* strains. Shown are the results of PCR-based SNP assays performed to elucidate the phylogenetic position of strains. Indicated at the top of the column is the whole strain pool of *B. anthracis* genotype A.Br. 008/011; the vertical black line indicates the assays in a direction of revealing increasing proximity to the heroin-associated strains. SNPs common to those of some strains from Turkey (*2*) are shown in light gray, and SNPs unique for the heroin-associated strains (*2*), including the isolate of 2000 from Norway, are depicted in dark gray (SNP designations and alleles are indicated).

This reemergence of drug-related anthrax in Europe strengthens the view that heroin may provide a continuing route of entry for *B. anthracis* into Western Europe (*2*). Ideally, this unfortunate deadly incident could offer an opportunity to sensitize heroin users to the risks for severe infection and to educate public health officials to be vigilant for this rare disease. This study also shows the power of molecular genotyping approaches for trace-back analysis of infectious disease agents.
